# A family-based genome-wide association study of chronic rhinosinusitis with nasal polyps implicates several genes in the disease pathogenesis

**DOI:** 10.1371/journal.pone.0185244

**Published:** 2017-12-18

**Authors:** Anton Bohman, Julius Juodakis, Martin Oscarsson, Jonas Bacelis, Mats Bende, Åsa Torinsson Naluai

**Affiliations:** 1 Department of Otorhinolaryngology, Uppsala University Hospital, Uppsala, Sweden; 2 Department of Obstetrics and Gynecology, Institute of Clinical Sciences, Sahlgrenska Academy, University of Gothenburg, Gothenburg, Sweden; 3 Department of Otorhinolaryngology, Skaraborg Hospital, Skövde, Sweden; 4 Department of Microbiology and Immunology, Institute of Biomedicine, Sahlgrenska Academy, University of Gothenburg, Gothenburg, Sweden; Beijing Tongren Hospital, CHINA

## Abstract

**Background:**

The pathogenesis of chronic rhinosinusitis with nasal polyps is largely unknown. Previous studies have given valuable information about genetic variants associated with this disease but much is still unexplained. Our goal was to identify genetic markers and genes associated with susceptibility to chronic rhinosinusitis with nasal polyps using a family-based genome-wide association study.

**Methods:**

427 patients (293 males and 134 females) with CRSwNP and 393 controls (175 males and 218 females) were recruited from several Swedish hospitals. SNP association values were generated using DFAM (implemented in PLINK) and Efficient Mixed Model Association eXpedited (EMMAX). Analyses of pathway enrichment, gene expression levels and expression quantitative trait loci were then performed in turn.

**Results:**

None of the analysed SNPs reached genome wide significant association of 5.0 x 10−^8^. Pathway analyses using our top 1000 markers with the most significant association p-values resulted in 138 target genes. A comparison between our target genes and gene expression data from the NCBI Gene Expression Omnibus database showed significant overlap for 36 of these genes. Comparisons with data from expression quantitative trait loci showed the most skewed allelic distributions in cases with chronic rhinosinusitis with nasal polyps compared with controls for the genes *HLCS*, *HLA-DRA*, *BICD2*, *VSIR* and *SLC5A1*.

**Conclusion:**

Our study indicates that *HLCS*, *HLA-DRA*, *BICD2*, *VSIR* and *SLC5A1* could be involved in the pathogenesis of chronic rhinosinusitis with nasal polyps. *HLA-DRA* has been associated with chronic rhinosinusitis with nasal polyps in previous studies and *HLCS*, *BICD2*, *VSIR* and *SLC5A1* may be new targets for future research.

## Introduction

Chronic rhinosinusitis (CRS) as defined by the European Position Paper on Rhinosinusitis and Nasal Polyps (EPOS) [1] is classified into chronic rhinosinusitis with nasal polyps (CRSwNP) and chronic rhinosinusitis without nasal polyps (CRSsNP). CRSwNP is a disease characterized by benign outgrowths from the middle meatus of the nasal cavity and chronic sinonasal inflammation. CRSwNP is a common chronic disease and depending on the geographical area, 2–4% of the population is afflicted [2–4]. The disease causes individual suffering and a decreased quality of life [5,6]. Risk factors include asthma, male sex and increasing age. The disease often requires a combination of surgical and medical treatment. However, CRSwNP often recurs even after therapy.

The aetiology of the disease is unknown. Several environmental factors have been suggested and previous studies have also shown an increased prevalence among relatives [7,8] and a higher rate of positive family history of CRSwNP among those affected [9–11], confirming a genetic susceptibility to the disease. Compared to the general population, having an afflicted family member increases the risk of disease five times [7]. Genetic studies on patients with CRSwNP could help to explain the pathogenesis of the disease and over time identify new drug targets leading to a more effective, individually tailored, therapy.

Genetic association can be explored using candidate gene studies or genome-wide association studies (GWAS). Candidate gene studies usually investigate a small number of single-nucleotide polymorphisms (SNPs) or other types of genetic variation, in order to find or reject associations between the genetic variants and the disease in question. These studies rely on previous knowledge and hypotheses regarding which SNPs to suspect and investigate. In comparison, a GWAS investigates hundreds of thousands of SNPs across the whole genome and is therefore not reliant on previous knowledge or hypotheses regarding the pathogenesis of the investigated disease or trait. A large number of GWASs have been performed for various complex diseases such as diabetes and asthma which has led to the finding of novel genetic pathways [12]. There is currently no published GWAS performed only on patients with CRSwNP but there is a pooling-based GWAS done on patients with CRS (both CRSsNP and CRSwNP) [13] as well as several studies of candidate genes [14]. These studies have implicated several genes and pathways such as the *cystic fibrosis transmembrane conductance regulator gene* (*CFTR*) [15,16] and, among others, genes involved in immunity [17–20], inflammation [21,22], tissue remodelling [20] and arachidonic acid metabolism [23]

Even though GWA studies have been successful in detecting genetic variants associated with many common diseases, the inability to explain most of the estimated heritability makes linkage analysis an alternative to detect possible rare variants. To this date, no published linkage analysis have been performed on subjects with CRSwNP. However, one such study has been performed on 8 subjects with CRS (not specified whether any of them had CRSwNP) which found a linkage signal on chromosome 7q [24]. A combination of a GWAS and linkage analysis such as a family-based GWAS could be a more potent way of identifying both common and rare variants associated with CRSwNP [25].

### Aim

The aim of this study is to identify SNPs and genes associated with CRSwNP susceptibility using a family-based approach.

## Materials and methods

### Material

367 patients with CRSwNP (250 men and 117 women, mean age 52.3 years) from three Swedish ear, nose and throat clinics were recruited. These subjects were all known patients at their respective clinics, all of them fulfilled the EPOS criteria for CRSwNP [1] and had at least intermittently been on either intranasal or systemic corticosteroids, most of them had undergone at least one operation for the condition. In order to increase power level for genome-wide analysis, patients with associated diseases such as asthma or aspirin-exacerbated respiratory disease (AERD) were not excluded. A total of 453 first-degree relatives (218 men, 235 women, mean age 49.4 years) were also recruited.

The study was carried out in accordance with the Declaration of Helsinki and was approved by the Ethics Committee at the University of Gothenburg, Sweden. Written consent was obtained from all participants.

### Investigation

Nasal endoscopy was performed on all participants using a 2.7 mm rigid endoscope (KARL STORZ) and the participants were subsequently phenotyped as either having CRSwNP or being free from this disease. Additional data about asthma and corticosteroid medication (used to counter symptoms from either the upper or lower airways) was obtained via a structured interview. Peripheral blood samples were collected from each individual.

DNA was extracted from whole blood using an in-house protocol at KBiosciences (LGC Genomics, Hoddeston UK). The HiSeq Illumina platform was used for genotyping. 144 of the samples were run on Illumina Omni Express bead chips and the remaining 676 on the Illumina Core Exome array.

### Quality control

Individuals were removed if they showed >2% missing calls (all samples passed), heterozygosity >3 SDs above or below mean, >100 Mendelian errors). Markers with >2% missing calls, >3 Mendelian errors or those with minor allele frequency = 0 (i.e. monomorphic loci) were excluded.

SNPs with low linkage disequilibrium among each other (LD) (r^2^<0.2) were selected for population structure analysis. As a reference, samples from the 1000 Genomes project, Phase 3 were retrieved. Principal component analysis was then performed using GCTA, a genome-wide complex trait analysis software [26], and the first three principal components were investigated. As a measure of non-European admixture in each sample, we calculated the Euclidean distance *E* from that sample to the mass centre of the CEU reference group. Individuals with *E* > 5 *SD*_*E*_ were removed (2 samples).

Only autosomal markers shared in both genotyping platforms were retained. Finally, principal components analysis was performed to check for batch effects. Visual inspection of sample scores along the first three principal components showed no differences between batches.

### Association testing

Two methods were used to generate SNP association values. First, we used DFAM, implemented in PLINK, which combines the transmission disequilibrium test (TDT), the sibling TDT and an allelic test for unrelated cases and controls in a single Cochran-Mantel-Haenszel test for each marker [27]. The second method was EMMAX (Efficient Mixed Model Association eXpedited), implemented in Golden Helix SNP & Variation Suite v8.3.4 [28]. This method involves computing an empirical relatedness matrix of the samples, and using this relatedness as a covariate in linear regression for each marker [29]. We performed this test using additive, dominant and recessive models, and the smallest p-value from the three models was assigned to each SNP. A commonly used level of significance in conventional GWA-studies on unrelated subjects is 5x10^-8^ [30] but many variants that do not reach this level of significance can still be true disease-influencing variation. The decision was therefore made to perform post-GWAS analyses for the 1000 SNPs with the lowest p-values for each method rather than adhering to a strict threshold for the level of significance.

### Pathway enrichment analysis

The top 1000 markers with the most significant association p-values were combined into intervals of SNPs in high LD (defined by pairwise r^2^>0.25). This process was performed separately for DFAM and EMMAX association results.

INRICH software was then used to detect possible pathway enrichment within these regions [31]. To calculate the significance of such overlaps, the process was repeated 50,000 times with random genomic regions, however matched in size and SNP density. INRICH analysis was performed separately using DFAM or EMMAX results and Gene Ontology (GO) or Kyoto Encyclopedia of Genes and Genomes (KEGG)-based gene-sets. The 20 gene-sets with the highest enrichment p-values were retrieved from each of these setups. INRICH produced a list of genes which were located close to the top GWAS ‘hits’ in the genome and that share functional annotations. All genes retrieved in this way from the four INRICH analyses (DFAM+GO, EMMAX+GO, DFAM+KEGG, EMMAX+KEGG) were combined together, creating a list of target NP genes implicated in this study.

### Gene expression data

Publicly-available gene expression data, collected by Plager et al. [32], was retrieved from NCBI Gene Expression Omnibus (GEO) database ([33]; series accession number GSE23552). Per authors’ recommendation, two samples (aCRSm1 and aCRSm2) were excluded, leaving 20 case samples (all from patients with CRSwNP) and 17 control samples from either allergic rhinitis patients or healthy individuals. Expression levels between the case and control groups were compared using the GEO2R interface. All genes corresponding to probes with significant difference in expression levels (Benjamini-Hochberg FDR < 0.05) comprise the differentially-expressed gene set.

### Expression quantitative trait loci (eQTL) analysis

Two datasets were used for eQTL analysis: Blood eQTL from Westra et al. [34] and Multiple Tissue Human Expression Resource (MuTHER) project [35]. These datasets are produced by microarray genotyping and expression profiling of selected tissue samples. SNP variations are then associated with gene expression patterns, resulting in a list of regulatory SNPs for each gene.

In MuTHER project, the regulatory effects of each SNP were determined in adipose, skin tissues and lymphoblastoid cell lines (LCL). For each SNP-gene pair we have retained either LCL or skin data, corresponding to the tissue with more significant regulatory effect (i.e. lower p-value in skin samples meant that LCL data was discarded for that SNP-gene pair). We also excluded all SNPs with p-values > 0.05 or absolute effect size (regression coefficient β) of < 0.01. FDR of 0.5 was used as a cut-off for the Blood dataset, with no additional limits on effect size.

To check for directed eQTL enrichment, all eQTL SNPs for each gene of interest were extracted and classified according to the direction of their regulatory effect (up-regulating or down-regulating). The frequency of the allele bearing the reported regulatory effect was then determined in our GWAS cases and controls using PLINK [27]. The marker was then assigned to a bin depending on whether the regulatory allele shows higher frequency in cases or in controls. In this way, a 2x2 contingency table was constructed for each gene, where all SNPs fall into one of four bins (up-regulating + less frequent in cases; up-regulating + more frequent in cases; down-regulating + less frequent in cases; down-regulating + more frequent in cases). Fisher’s test was used to test whether the regulatory effect and frequency difference are dependent.

However, Fisher’s test does not account for the effect of LD between SNPs. Therefore, the empirical significance was calculated using an iterative procedure. Genes were ordered according to the number of eQTL SNPs remaining after all filters; genes found in the differentially-expressed NP set (as described in the previous section) were removed; for each gene of interest with *n* SNPs, 500 genes with the same number *n* of SNPs are retrieved; if less than 500 genes have the required number of SNPs, genes with *n*+1 (then *n*+2, *n*+3…) SNPs are also retrieved, and *n* SNPs are randomly selected for analysis in those genes. Each gene is analysed in the same manner as the target gene. Resulting empirical distribution of p-values is used to determine the empirical significance for the gene of interest.

**Fig 1:**
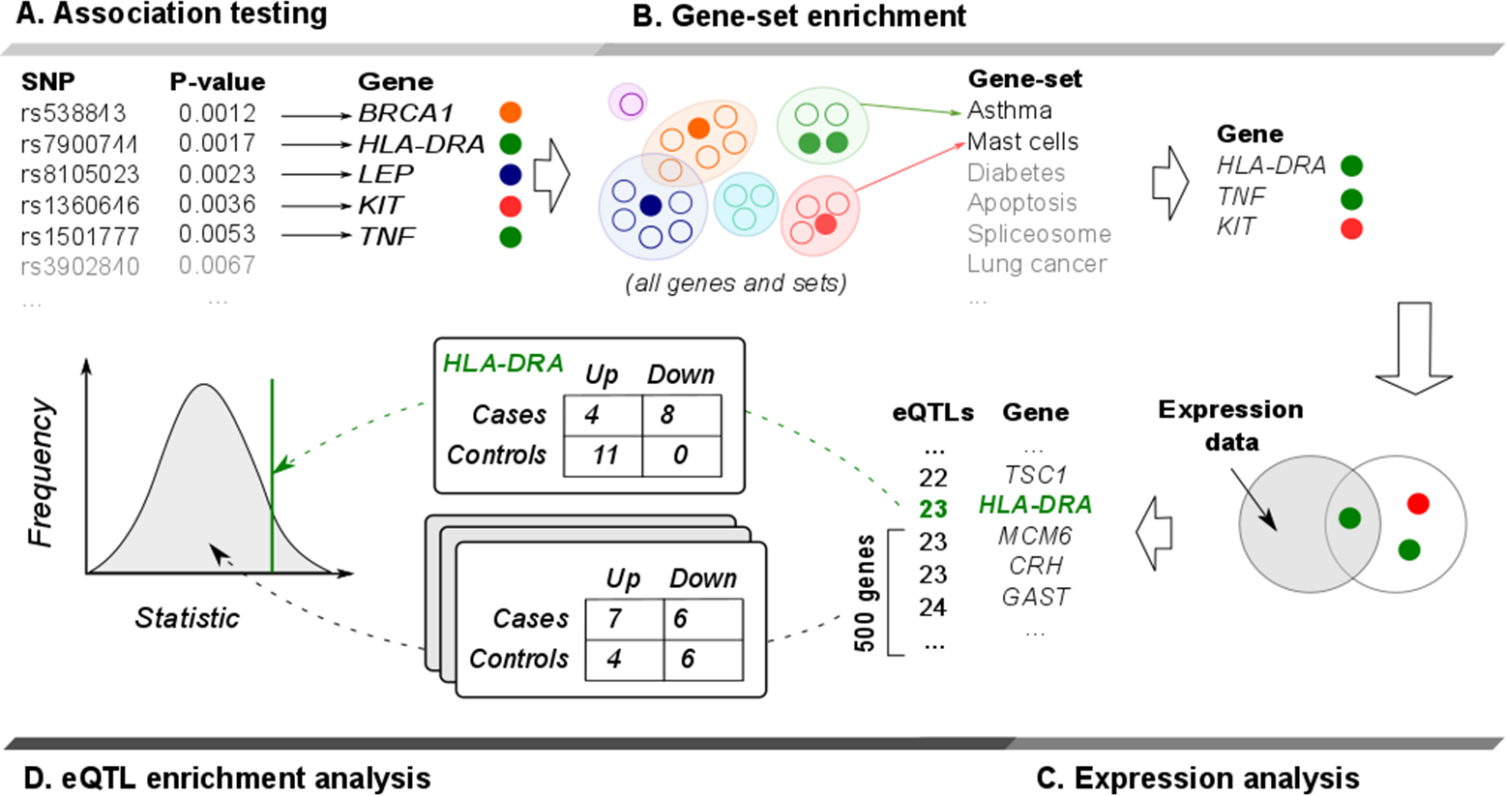
Workflow of analysis. A. Initially, SNP association p-values are produced by an association test. Based on these values, top 1000 SNPs are selected and annotated to nearby genes. B. INRICH software is then used to detect over-represented gene-sets (empty circles denote all genes that were not detected by GWAS). Genes from top 20 such sets are retrieved, and only the ones overlapping with GWAS hits are analysed further. C. Using publicly available expression data from NP samples, this gene list is filtered to retain only differentially expressed genes. D. For each of these remaining targets, known eQTLs are assigned into bins based on effect direction (up- or down- regulating) and frequency distribution in our genotyping data (higher frequency in cases or in controls). Fisher's exact test is used to evaluate the observed distribution. 500 genes with equal or higher count of eQTLs are analysed in the same way, and statistic values generated from this control set are then compared with the target gene statistic to estimate the empirical significance.

The workflow of the analysis is shown in Fig 1.

## Results

### Genetic analysis

Six samples were removed due to heterozygosity > 3 SDs above or below mean, three samples were removed due to > 100 Mendelian errors and one sample due to a mismatch between genotyped and indicated sex. The final dataset after quality-control consists of 782 individuals and 233 409 SNPs. Additional data about the subjects who passed quality control are presented in Table 1. Of the 406 individuals with nasal polyps, 350 were index patients with CRSwNP, 22 of the non-index patients knew they had polyps beforehand and 34 had newly discovered polyps.

**Table 1:** Descriptive statistics of the participants who passed quality control.

	Nasal polyps n = 406 Mean age 56.42, SD 14.3	Unaffected subjects n = 376 Mean age 47.85, SD 17.0	p-value
**Sex**	**275 male, 131 female**	**169 male, 207 female**	**<0.001**^**1**^
**Asthma**	**209 (51.5%)**	**65 (17.3%)**	**<0.001**^**1**^
**Use of corticosteroids for nasal symptoms**	**261 (64.3%)**	**24 (6.4%)**	**<0.001**^**1**^
**Use of corticosteroids for pulmonary symptoms**	**160 (39.4%)**	**30 (8%)**	**<0.001**^**1**^

^1^ Comparison of the prevalence/proportion between the groups by Chi-2 test.

**Fig 2:**
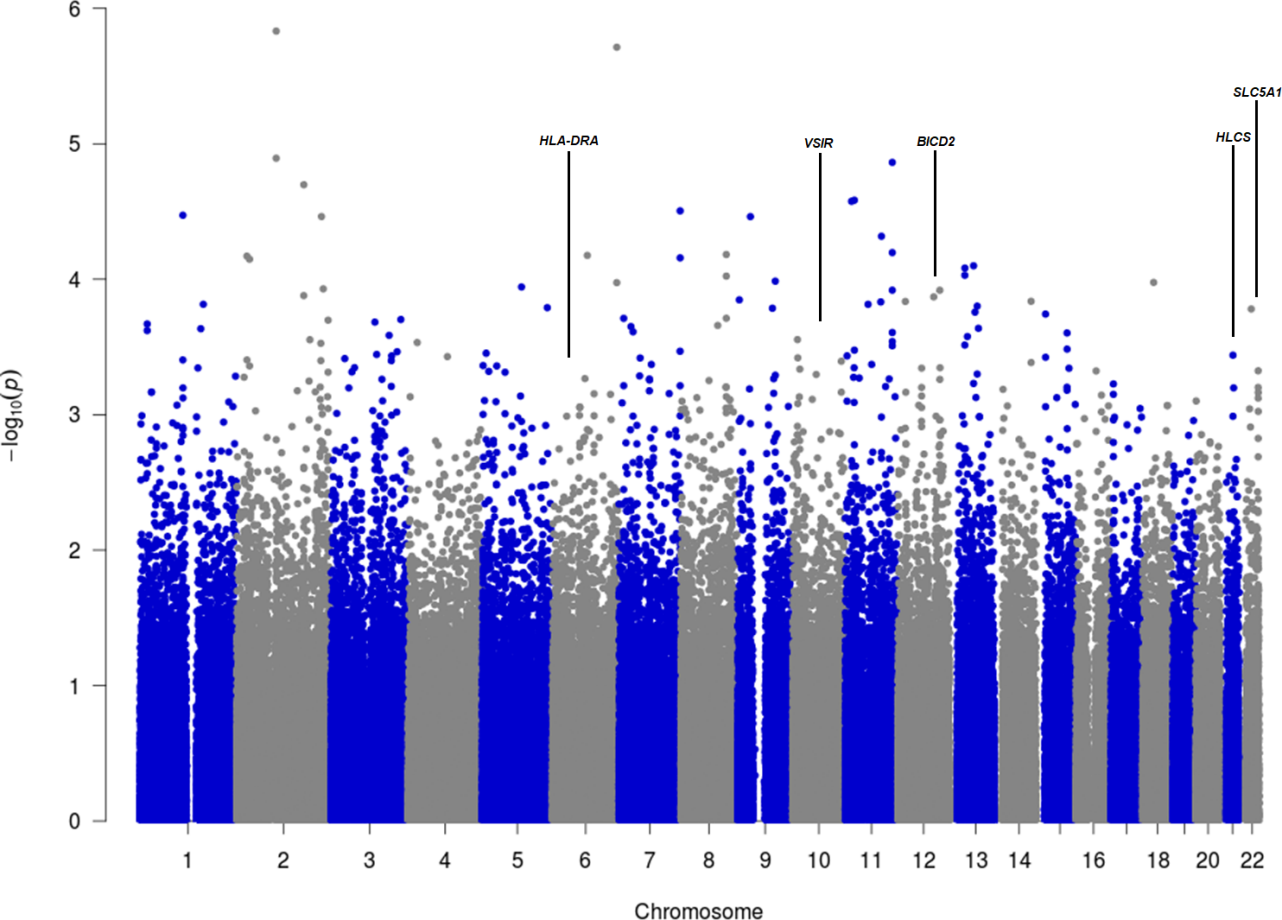
Manhattan plot from the DFAM analysis.

**Fig 3:**
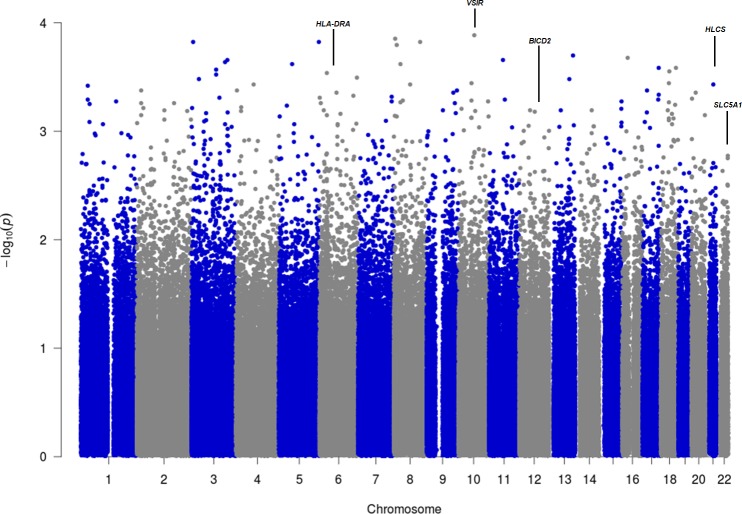
Manhattan plot from the EMMAX analysis.

The Manhattan plot from the DFAM analysis is provided in Fig 2 and the Manhattan plot from the EMMAX analysis in Fig 3. Table 2 lists the 30 SNPs with the strongest associations from the DFAM analysis and Table 3 lists the 30 SNPs with the strongest association values from the EMMAX analysis. None of tested SNPs reached a significance of 5x10^-8^, the top ranking SNP from the DFAM analysis was rs4629180 with a p-value of 1.47x10^-6^, the top ranking SNP from the EMMAX analysis was rs2491026 with a p-value of 0.00014.

**Table 2:** Top 30 associated SNPs from DFAM analysis.

SNP	CHR	BP	A1	A2	p	OBS	EXP
**rs4629180**	**2**	**102088370**	**G**	**A**	**1.47E-06**	**107**	**125.1**
**rs227457**	**6**	**165469641**	**G**	**T**	**1.94E-06**	**140**	**159.8**
**rs4851455**	**2**	**102083183**	**T**	**C**	**1.28E-05**	**124**	**141.3**
**rs10790443**	**11**	**121234131**	**C**	**A**	**1.37E-05**	**154**	**137.2**
**rs4531900**	**2**	**173125108**	**C**	**T**	**2.01E-05**	**199**	**181.2**
**rs7941380**	**11**	**23660635**	**C**	**T**	**2.61E-05**	**110**	**125.9**
**rs7932807**	**11**	**16438411**	**G**	**A**	**2.66E-05**	**176**	**157.9**
**rs9654699**	**7**	**157512750**	**G**	**A**	**3.13E-05**	**114**	**129.5**
**rs343791**	**1**	**111301623**	**T**	**C**	**3.37E-05**	**147**	**162.6**
**rs744564**	**2**	**218588959**	**A**	**C**	**3.45E-05**	**63**	**51.7**
**rs1326917**	**9**	**32717956**	**A**	**G**	**3.45E-05**	**155**	**172.6**
**rs2399685**	**11**	**93295334**	**C**	**T**	**4.82E-05**	**175**	**158.7**
**rs72991**	**11**	**121243716**	**C**	**T**	**6.36E-05**	**133**	**118.3**
**rs10086506**	**8**	**116988947**	**G**	**T**	**6.57E-05**	**182**	**165.8**
**rs205226**	**6**	**89743077**	**C**	**T**	**6.67E-05**	**163**	**179.8**
**rs1275928**	**2**	**26865646**	**C**	**A**	**6.75E-05**	**171**	**188.8**
**rs10253909**	**7**	**157516956**	**C**	**A**	**6.96E-05**	**159**	**174.5**
**rs921705**	**2**	**33474645**	**C**	**T**	**7.11E-05**	**144**	**161.1**
**rs301662**	**13**	**61446809**	**T**	**G**	**7.96E-05**	**42**	**31.83**
**rs9548374**	**13**	**39077255**	**T**	**C**	**8.27E-05**	**135**	**151.9**
**rs6563618**	**13**	**39021562**	**A**	**G**	**9.35E-05**	**134**	**150.2**
**rs2223063**	**8**	**117050089**	**C**	**T**	**9.49E-05**	**158**	**143.4**
**rs1057713**	**9**	**96714161**	**G**	**A**	**1.03E-04**	**147**	**164.1**
**rs8096598**	**18**	**28584774**	**C**	**A**	**1.06E-04**	**24**	**32.5**
**rs9364788**	**6**	**165458279**	**C**	**A**	**1.06E-04**	**105**	**120.7**
**rs10065655**	**5**	**101192693**	**C**	**T**	**1.14E-04**	**133**	**118.9**
**rs12053273**	**2**	**223106526**	**A**	**G**	**1.18E-04**	**103**	**88.87**
**rs10750191**	**11**	**121239305**	**G**	**A**	**1.21E-04**	**130**	**115.8**
**rs10507221**	**12**	**108373750**	**C**	**T**	**1.21E-04**	**87**	**75.17**
**rs2016394**	**2**	**172972971**	**A**	**G**	**1.32E-04**	**192**	**208.4**

CHR: Chromosome

BP: Base-pair, position of the SNP on the chromosome

A1: Allele 1

A2: Allele 2

p: p-value

OBS: Observed number of CRSwNP patients with allele 1

EXP: Expected number of CRSwNP patients with allele 1 under the assumption of random inheritance of alleles.

Beta: Effect size

Model: The model of inheritance that produced the smallest p-value in the analysis; ADD = additive, DOM = dominant, REC = recessive

**Table 3:** Top 30 associated SNPs from EMMAX analysis.

SNP	CHR	BP	A1	A2	p	Beta	Model
**rs2491026**	**10**	**70802309**	**A**	**G**	**0.00014**	**-0.40**	**REC**
**rs3824310**	**8**	**6386250**	**C**	**T**	**0.00014**	**-0.22**	**REC**
**rs712776**	**3**	**7463316**	**T**	**C**	**0.00015**	**0.26**	**REC**
**rs7713983**	**5**	**177690978**	**T**	**C**	**0.00015**	**0.24**	**REC**
**rs799855**	**8**	**117265106**	**C**	**T**	**0.00016**	**0.44**	**REC**
**rs1671389**	**8**	**13181518**	**A**	**C**	**0.00017**	**0.49**	**REC**
**rs9670370**	**13**	**105738427**	**T**	**C**	**0.00021**	**0.40**	**REC**
**rs246334**	**16**	**23738355**	**C**	**T**	**0.00021**	**0.14**	**DOM**
**rs2366408**	**3**	**159696099**	**T**	**G**	**0.00022**	**0.10**	**ADD**
**rs10897316**	**11**	**62789763**	**G**	**A**	**0.00023**	**-0.16**	**REC**
**rs6501658**	**17**	**71661629**	**C**	**T**	**0.00023**	**0.11**	**ADD**
**rs9821781**	**3**	**149362405**	**G**	**A**	**0.00024**	**-0.14**	**DOM**
**rs7459662**	**8**	**29682635**	**C**	**A**	**0.00024**	**-0.21**	**REC**
**rs294482**	**5**	**58845915**	**G**	**T**	**0.00024**	**0.37**	**REC**
**rs1372771**	**18**	**67153636**	**G**	**A**	**0.00025**	**0.29**	**REC**
**rs619878**	**3**	**109677974**	**G**	**A**	**0.00027**	**-0.28**	**REC**
**rs1941476**	**18**	**37686439**	**C**	**T**	**0.00029**	**0.13**	**DOM**
**rs660895**	**6**	**32577380**	**G**	**A**	**0.00029**	**0.23**	**REC**
**rs9860143**	**3**	**109708722**	**A**	**G**	**0.00030**	**-0.16**	**ADD**
**rs6920797**	**6**	**166237461**	**G**	**T**	**0.00033**	**0.18**	**REC**
**rs12584112**	**13**	**89159334**	**T**	**C**	**0.00034**	**0.13**	**DOM**
**rs1127898**	**3**	**33186356**	**C**	**T**	**0.00034**	**0.11**	**ADD**
**rs870391**	**18**	**35885805**	**T**	**C**	**0.00037**	**0.22**	**REC**
**rs7086393**	**10**	**58250470**	**G**	**A**	**0.00037**	**0.16**	**REC**
**rs10957577**	**8**	**72919087**	**G**	**A**	**0.00037**	**0.18**	**REC**
**rs355687**	**4**	**78505578**	**C**	**T**	**0.00037**	**-0.13**	**DOM**
**rs2211678**	**21**	**33339340**	**T**	**C**	**0.00038**	**-0.23**	**REC**
**rs12736883**	**1**	**31241312**	**A**	**G**	**0.00039**	**-0.14**	**DOM**

CHR: Chromosome

BP: Base-pair, position of the SNP on the chromosome

A1: Allele 1

A2: Allele 2

p: p-value

OBS: Observed number of CRSwNP patients with allele 1

EXP: Expected number of CRSwNP patients with allele 1 under the assumption of random inheritance of alleles.

Beta: Effect size

Model: The model of inheritance that produced the smallest p-value in the analysis; ADD = additive, DOM = dominant, REC = recessive

From the pathway enrichment analysis we extracted the top 20 gene-sets from each of the four INRICH analyses resulting in a combined list of 138 target CRSwNP genes from this study (S1 Table).

Out of our 138 target genes from the INRICH analysis, 36 genes showed a significant difference in mRNA expression levels between nasal polyp tissue and normal tissue (from Plager et al. [32]) (Table 4)

**Table 4:** Overlap between genes from INRICH analysis and expression data (from Plager et al. [32]).

Name	ENTREZ ID	Source	Probe ID	Adjusted p	Log2FC	CHR	FC
***IL2RA***	**3559**	**GO + EMMAX**	**3275729**	**3.87e-09**	**2.25**	**10**	**4.75**
***PDGFD***	**80310**	**GO + EMMAX**	**3389077**	**1.26e-05**	**0.83**	**11**	**1.78**
***P4HA1***	**5033**	**GO + DFAM**	**3294159**	**0.0035**	**0.74**	**10**	**1.67**
***HLA-DRA***	**3122**	**KEGG + EMMAX**	**2903189**	**0.0082**	**0.69**	**6**	**1.61**
***LAMB3***	**3914**	**KEGG + EMMAX**	**2453793**	**0.0084**	**0.63**	**1**	**1.55**
***VSIR***	**64115**	**GO + DFAM**	**3293724**	**0.0002**	**0.63**	**10**	**1.55**
***TIAM1***	**7074**	**KEGG + DFAM**	**3928668**	**0.0062**	**0.44**	**21**	**1.36**
***E2F3***	**1871**	**KEGG + EMMAX**	**2897576**	**0.0060**	**0.44**	**6**	**1.36**
***SIPA1***	**6494**	**GO + DFAM**	**3335465**	**0.0027**	**0.42**	**11**	**1.34**
***LYN***	**4067**	**KEGG + DFAM**	**3098977**	**0.0099**	**0.40**	**8**	**1.32**
***CACNA1D***	**776**	**KEGG + DFAM**	**2624385**	**0.014**	**0.37**	**3**	**1.30**
***CPLX2***	**10814**	**GO + DFAM**	**2842255**	**0.0091**	**0.35**	**5**	**1.28**
***COL16A1***	**1307**	**GO + EMMAX**	**2404546**	**0.0071**	**0.30**	**1**	**1.23**
***PPARD***	**5467**	**GO + EMMAX**	**2904597**	**0.0032**	**0.29**	**6**	**1.23**
***BICD2***	**23299**	**GO + EMMAX**	**3214984**	**0.030**	**0.25**	**9**	**1.19**
***STXBP1***	**6812**	**GO + DFAM**	**3189932**	**0.031**	**-0.28**	**9**	**0.83**
***DOCK1***	**1793**	**KEGG + DFAM**	**3269939**	**0.016**	**-0.29**	**10**	**0.82**
***GNG12***	**55970**	**KEGG + DFAM**	**2417272**	**0.037**	**-0.34**	**1**	**0.79**
***CAPN2***	**824**	**GO + EMMAX**	**2382117**	**0.032**	**-0.35**	**1**	**0.79**
***USP13***	**8975**	**GO + DFAM**	**2654091**	**0.015**	**-0.35**	**3**	**0.79**
***ADCY1***	**107**	**KEGG + EMMAX**	**3000342**	**0.0004**	**-0.37**	**7**	**0.78**
***NMD3***	**51068**	**KEGG + EMMAX**	**2650538**	**0.032**	**-0.37**	**3**	**0.78**
***ANK2***	**287**	**GO + DFAM**	**2740067**	**0.0026**	**-0.37**	**4**	**0.77**
***OGFOD1***	**55239**	**GO + DFAM**	**3692928**	**0.022**	**-0.38**	**16**	**0.77**
***NDUFS5***	**4725**	**KEGG + EMMAX**	**2331178**	**0.019**	**-0.43**	**1**	**0.74**
***CPEB3***	**22849**	**GO + EMMAX**	**3300242**	**2.22e-05**	**-0.49**	**10**	**0.71**
***LRP5***	**4041**	**GO + EMMAX**	**3337516**	**0.0006**	**-0.50**	**11**	**0.71**
***HLCS***	**3141**	**KEGG + DFAM**	**3931112**	**1.09e-05**	**-0.51**	**21**	**0.70**
***MYL9***	**10398**	**KEGG + EMMAX**	**3883921**	**0.0034**	**-0.58**	**20**	**0.70**
***PLCB1***	**23236**	**KEGG + EMMAX**	**3875642**	**0.0013**	**-0.67**	**20**	**0.63**
***FERMT2***	**10979**	**GO + EMMAX**	**3564919**	**0.0002**	**-0.70**	**14**	**0.62**
***CHRM3***	**1131**	**GO + EMMAX**	**2387606**	**0.0002**	**-0.73**	**1**	**0.60**
***LEPR***	**3953**	**GO + EMMAX**	**2340433**	**0.0022**	**-0.76**	**1**	**0.59**
***PDE3A***	**5139**	**GO + EMMAX**	**3407453**	**1.04e-06**	**-1.16**	**12**	**0.45**
***LYZ***	**4069**	**KEGG + EMMAX**	**3421511**	**1.16e-06**	**-1.46**	**12**	**0.36**
***SLC5A1***	**6523**	**KEGG + DFAM**	**3943234**	**4.32e-05**	**-1.62**	**22**	**0.33**

Name: HUGO gene ID

ENTREZ ID: gene ID from ENTREZ

Source: combination of pathways and methods that implicated this gene, GO = Gene Ontology, KEGG = Kyoto Encyclopedia of Genes and Genomes

Probe ID: ID of the corresponding probe from the expression dataset

Adjusted p: FDR adjustment

Log2FC: log2 fold change; 0 means no change, positive means up-regulation in NP samples, negative means down-regulation

CHR: chromosome

FC: fold change (1 means no change)

Finally, the eQTL analysis for the Blood eQTL dataset showed significantly skewed distributions of eQTLs in cases with CRSwNP compared to controls for *HLCS* (empirical p-value 0.014) *HLA-DRA* (empirical p-value 0.02) and *BICD2* (empirical p 0.046) (Table 5). The same analysis performed with MuTHER eQTL dataset showed significantly skewed eQTL distribution in cases for *VSIR* (empirical p-value 0.006), *HLCS* (empirical p-value 0.014) and *BICD2* (empirical p-value 0.016). *SLC5A1* also had a skewed distribution with an empirical p-value of 0.052, these results are provided in Table 6.

**Table 5:** Results from the eQTL analysis, Blood dataset.

Name	p	Empirical p	Log2FC	SNPs	Up +	Up -	Down +	Down -
***HLCS***	**0.00068**	**0.014**	**-0.51**	**22**	**1**	**17**	**4**	**0**
***HLA-DRA***	**0.0014**	**0.020**	**0.69**	**23**	**4**	**8**	**11**	**0**
***BICD2***	**0.0082**	**0.046**	**0.24**	**16**	**0**	**10**	**4**	**2**
***PDGFD***	**0.0065**	**0.066**	**0.83**	**18**	**0**	**2**	**16**	**0**
***NDUFS5***	**0.0098**	**0.080**	**-0.43**	**20**	**8**	**1**	**3**	**8**
***TIAM1***	**0.048**	**0.11**	**0.44**	**10**	**5**	**0**	**1**	**4**
***VSIR***	**0.027**	**0.16**	**0.63**	**24**	**7**	**1**	**5**	**11**
***LYZ***	**0.068**	**0.27**	**-1.46**	**32**	**4**	**14**	**8**	**6**
***LEPR***	**0.36**	**0.52**	**-0.76**	**44**	**13**	**13**	**6**	**12**
***OGFOD1***	**1**	**0.76**	**-0.13**	**13**	**2**	**2**	**4**	**5**
***SIPA1***	**1**	**0.83**	**0.42**	**26**	**1**	**3**	**9**	**13**

Name: HUGO gene ID

p: unadjusted p-value, produced by Fisher's test for the target gene

Empirical p: p-value, calculated from the cumulative distribution of Fisher's test p-values for similar genes

Log2FC: log2 fold change; 0 means no change, positive means up-regulation in NP samples, negative means down-regulation

SNPs: number of eQTL SNPs tested in this analysis

Up +, Up -, Down + and Down—form the 2x2 contingency table for Fisher's test: Up +: number of up-regulating SNPs that show increased frequency in cases

Up -: number of up-regulating SNPs that show similar or decreased frequency in cases

Down +: number of down-regulating SNPs that show increased frequency in cases

Down -: number of down-regulating SNPs that show similar or decreased frequency in cases

**Table 6:** Results from the eQTL analysis, MuTHER dataset.

Name	p	Empirical p	Log2FC	SNPs	Up +	Up -	Down +	Down -
***VSIR***	**2.51e-05**	**0.006**	**0.63**	**51**	**25**	**1**	**5**	**10**
***HLCS***	**3.88e-05**	**0.014**	**-0.51**	**58**	**11**	**23**	**21**	**3**
***BICD2***	**0.0016**	**0.017**	**0.24**	**33**	**3**	**14**	**12**	**4**
***SLC5A1***	**0.0055**	**0.052**	**-1.62**	**25**	**3**	**5**	**16**	**1**
***PDGFD***	**0.0067**	**0.056**	**0.83**	**35**	**5**	**12**	**14**	**4**
***CAPN2***	**0.10**	**0.11**	**-0.35**	**13**	**2**	**4**	**6**	**1**
***LYN***	**0.027**	**0.14**	**0.40**	**29**	**4**	**12**	**9**	**4**
***PDE3A***	**0.14**	**0.16**	**-1.57**	**14**	**1**	**5**	**5**	**3**
***OGFOD1***	**0.034**	**0.17**	**-0.13**	**60**	**13**	**16**	**23**	**8**
***PLCB1***	**0.055**	**0.21**	**-0.67**	**40**	**15**	**6**	**7**	**12**
***FERMT2***	**0.11**	**0.25**	**-0.70**	**26**	**4**	**8**	**10**	**4**
***ADCY1***	**0.16**	**0.34**	**-0.37**	**52**	**18**	**11**	**9**	**14**
***HLA-DRA***	**0.32**	**0.37**	**0.69**	**18**	**8**	**2**	**4**	**4**
***TIAM1***	**0.23**	**0.41**	**0.44**	**46**	**18**	**9**	**9**	**10**
***STXBP1***	**0.39**	**0.41**	**-0.28**	**22**	**3**	**8**	**6**	**5**
***LRP5***	**0.31**	**0.42**	**-0.50**	**35**	**4**	**11**	**9**	**11**
***NDUFS5***	**0.39**	**0.44**	**-0.43**	**24**	**11**	**3**	**6**	**4**
***IL2RA***	**0.40**	**0.49**	**2.25**	**51**	**16**	**10**	**12**	**13**
***ANK2***	**0.46**	**0.59**	**-0.37**	**30**	**6**	**10**	**8**	**6**
***COL16A1***	**0.67**	**0.66**	**0.30**	**23**	**7**	**7**	**3**	**6**
***LAMB3***	**0.54**	**0.68**	**0.63**	**43**	**12**	**6**	**14**	**11**
***CPEB3***	**0.72**	**0.71**	**-0.49**	**39**	**16**	**12**	**5**	**6**
***GNG12***	**1**	**0.73**	**-0.34**	**16**	**1**	**3**	**4**	**8**
***CACNA1D***	**1**	**0.76**	**0.37**	**23**	**3**	**5**	**6**	**9**
***CPLX2***	**0.70**	**0.79**	**0.17**	**26**	**8**	**8**	**4**	**6**
***DOCK1***	**1**	**0.79**	**-0.29**	**34**	**7**	**7**	**11**	**9**
***PPARD***	**0.75**	**0.81**	**0.29**	**39**	**11**	**8**	**10**	**10**
***LEPR***	**1**	**0.88**	**-0.76**	**74**	**18**	**19**	**17**	**20**

Name: HUGO gene ID

p: unadjusted p-value, produced by Fisher's test for the target gene

Empirical p: p-value, calculated from the cumulative distribution of Fisher's test p-values for similar genes

Log2FC: log2 fold change; 0 means no change, positive means up-regulation in NP samples, negative means down-regulation

SNPs: number of eQTL SNPs tested in this analysis

Up +, Up -, Down + and Down—form the 2x2 contingency table for Fisher's test:

Up +: number of up-regulating SNPs that show increased frequency in cases

Up -: number of up-regulating SNPs that show similar or decreased frequency in cases

Down +: number of down-regulating SNPs that show increased frequency in cases

Down -: number of down-regulating SNPs that show similar or decreased frequency in cases

## Discussion

None of the SNPs in this study reached the suggested genome-wide significance level of 5x10^-8^. However, by using pathway enrichment and post-GWAS analyses, we identified five interesting genes that could be involved in the pathogenesis of CRSwNP in this study: *HLCS*, *HLA-DRA*, *BICD2*, *VSIR* and *SLC5A1*. Of these, only *HLA-DRA* has been presented in previous studies on subjects with CRSwNP [36].

The present study is the first GWAS performed only on subjects with CRSwNP. Using family relationship data and non-transmitted genetic variation for control, as in the TDT, is both a strength and a potential weakness. In most chromosomal regions, we expect the related individuals to be more similar compared with completely unrelated controls. Therefore, when there are differences between related individuals these are more likely to be due to the disease than to general differences in a population (population stratification). In practice this is a strength because it would be expected to lead to less false positive results. However, due to the increased risk of CRSwNP among relatives [7] and the increased prevalence of polyps with higher age [4], some of the relatives who we have defined as not having polyps could develop CRSwNP later in life and therefore be falsely classified as controls in this study. This could possibly have led to missed markers and genes of potential importance. However, most of the relatives in this study are middle-aged or older (mean age 49.4 years) and the prevalence of nasal polyps among them is 13% which makes it unlikely that more than a few percent of the relatives are falsely classified as phenotype negative. One could also argue that there could be subjects with asymptomatic polyps among the 55 relatives who had nasal polyps during endoscopy. However, 21 of them knew they had polyps beforehand and only 34 were unaware of this. The heritability of CRSwNP makes it much more likely that first-degree relatives of patients with CRSwNP would inherit variants associated with CRSwNP than inherit variants associated with asymptomatic nasal polyps. In the event that there are many relatives with polyps but without the predisposition to develop CRS it would indeed influence the GWAS findings, however excluding relatives with polyps would most likely influence the result in a more negative way and severely limit the study.

The *HLCS* gene was the most significant gene from the eQTL analysis in the Blood dataset and the second most significant in the MuTHER dataset. *HLCS* is under-expressed in nasal polyp tissue with significantly increased frequency of down-regulating alleles among cases in the eQTL analysis from both the MuTHER and the Blood datasets. This gene encodes the enzyme Holocarboxylase synthetase, which is important for biotin metabolism. Holocarboxylase synthetase itself has not been implicated in CRSwNP but a study from 2013 showed that the enzyme catalyses biotinylation of heat shock protein 72 thereby inducing the expression of the gene *RANTES (regulated on activation normal T-expressed and presumably secreted)* [37]. *RANTES* is implicated in multiple studies of CRSwNP; for example a study by Chao et al. found a positive correlation between plasma RANTES protein levels and severity of disease among patients with CRSwNP[38]. RANTES protein has also been detected in nasal polyps using immunological staining [39]. Although it seems counter-intuitive that *HLCS* is under-expressed when RANTES protein levels are higher in polyp tissue compared with controls, the up-regulation of *RANTES* might be a counter reaction to initially too low levels during an early disease phase. Further studies are needed to increase the knowledge about the role of *HLCS* in the pathogenesis of CRSwNP.

*HLA-DRA* is over-expressed in polyp tissue and has a significantly skewed distribution of eQTLs from the Blood dataset where cases have an increased number of down-regulating alleles. *HLA-DRA* is one of the major histocompatibility complex, class II genes. Polymorphisms in this gene have been associated with the presence of nasal polyps in asthmatic patients [36]. Additionally, polymorphisms in other *HLA class II* genes have been linked to CRSwNP [18,40]. Using HLA typing on a series of 29 patients with nasal polyps, with or without asthma, Moloney and Oliver found a significant increase in the haplotype AI/B8 in patients with both nasal polyps and asthma[41].

*BICD2* is over-expressed in nasal polyps and up-regulating alleles more common in controls and down regulating slightly more common in cases. The gene product is bicaudal D homolog 2, which has been shown to induce microtubule movement [42]. It is also linked to dominant congenital spinal muscular atrophy [43].

The relationship between gene-expression and eQTLs is reversed for *HLA-DRA* and *BICD2* where cases have an increased number of down-regulating eQTLs even though the gene is over-expressed in polyp tissue. Over-production in the diseased state could possibly be the result of the body compensating for an under-production in the pre-disease state, which hypothetically could have contributed to the development of the disease.

*VSIR (V-set immunoregulatory receptor)* is over-expressed in polyp tissue and up-regulating alleles from the MuTHER dataset are more common in our cases with CRSwNP compared with unaffected individuals. The gene codes for the protein V-type immunoglobulin domain-containing suppressor of T-cell activation, a member of the Ig superfamily. An experimental study has suggested that it could facilitate tumour invasiveness by regulating cell surface membrane-type 1 matrix metalloproteinase [44]. Lines et al. published an article showing that it also acts a negative checkpoint regulator that suppresses T cell activation [45]. It has not been implicated in CRSwNP in previous studies.

Even though the empirical p-value is slightly higher than 0.05 (empirical p-value 0.052) this study also implicates *SLC5A1* as borderline significant. *SLC5A1* is under-expressed in polyp tissue and also has an increased frequency of down-regulating alleles in our CRSwNP cases. The gene-product, solute carrier family 5 (sodium/glucose cotransporter) member 1 (SGLT1), is part of a family of sodium-dependent glucose transporters. Once again, this gene has not been associated with CRSwNP but one article suggests a positive substrate cross-regulation of SGLT1 and *CFTR* [46]. The *CFTR* gene is highly associated with cystic fibrosis, which often has CRSwNP as one of its clinical features [47]. Furthermore, a study from Varon et al. found an association between CRSwNP (without any other clinical features of cystic fibrosis) and mutations in the *CFTR* locus [48].

In order to reach a power level necessary for genome-wide analysis we decided to include all patients with CRSwNP regardless of any other diseases which could be associated to this condition. Another reason for this is that the only largely accepted subgrouping of CRS is the division into either CRSsNP or CRSwNP. This is in all likelihood a gross simplification of the pathophysiological and genetic mechanisms behind these conditions and CRS as defined by EPOS is probably a result of a large number of different sub-diseases, each with their own genetic and/or environmental background of which little is known at the present. In this study we did not exclude participants based on potential subgroups since there is still uncertainty surrounding a division into subgroups other than CRSsNP and CRSwNP and we chose to focus on the phenotype CRSwNP itself. Similarly, we chose not to record allergy history due to the controversy surrounding allergy as a possible association to CRSwNP [49]. However, two possible genetic subgroups of CRSwNP; CRSwNP with concomitant asthma and aspirin-exacerbated respiratory disease (AERD) warrant attention in our minds.

Since 51.5% of the participants with nasal polyps also had asthma it is possible that some of the associations could be due to an association with asthma. However, this is less likely since 17.3% of the subjects in the healthy group without polyps also had asthma. The high number of participants with both CRSwNP and asthma could have diluted the association analysis if their SNP-profiles differ significantly from subjects with CRSwNP but without asthma and potentially have made us miss markers of importance in the association analysis. This situation is likely to be countered by the gene enrichment and pathway analyses. *HLCS*, *BICD2*, and *SLC5A1* have not been connected to asthma, *VSIR* was implicated with lung function decline in non-asthmatic patients in a genome-wide study published in 2012 but this association could not be confirmed by replication [50]. *HLA-DRA* is associated to asthma [51] but also, as mentioned above, associated to the presence of nasal polyps in a cohort of asthmatic patients [36].

AERD is another condition linked to CRSwNP, a meta-analysis published in 2015 found a large variation in the prevalence of AERD among patients with CRSwNP among the included studies, the overall prevalence was 9.7% [52]. One of the included studies is from the same geographical region as 96% of our test subjects and found that the prevalence of AERD among subjects with CRSwNP was 6/82 but the numbers were thought to be too small for any meaningful statistical analysis [53]. Although some of our participants probably suffer from AERD, the overall number is in all likelihood too small to influence the association analysis and post-GWAS analyses significantly. *HLCS*, *BICD2*, *SLC5A1* and *VSIR* have not been associated with AERD in previous studies. *HLA-DRA* has not been linked to AERD but other *HLA class II* genes have [54], however, the same study that linked *HLA-DRA* to the presence of nasal polyps among asthmatic patients found two HLA-DRA polymorphisms to be potential markers for nasal polyp development in aspirin-tolerant asthma compared to the AERD subgroup [36].

With these caveats in mind, this study is the first of its kind. It is currently the only GWAS performed on CRSwNP and the only study that explores linkage and family-based genome-wide association with regards to this condition. Despite the issue of accurate phenotyping discussed above, this study suggests four novel genes as potential targets of interest for future research as well as once again implicate HLA-DRA.

### Conclusion

This study suggests that *HLA-DRA* as well as four additional genes; *HLCS*, *VSIR*, *BICD2* and *SLC5A1*, which have not been previously identified as associated with chronic rhinosinusitis with nasal polyps, could be important for the development of this disease.

## Supporting information

S1 Table List of target genes from the top 20 gene-sets in the INRICH analysis. (DOCX)

## Abbreviations

CRS: Chronic rhinosinusitis
CRSwNP: Chronic rhinosinusitis with nasal polyps
CRSsNP: Chronic rhinosinusitis without nasal polyps
AERD: Aspirin-exacerbated respiratory disease
EPOS: European Position Paper on Rhinosinusitis and Nasal Polyps
GWAS: Genome-wide association study
CFTR: Cystic fibrosis transmembrane regulator gene
SNP: Single-nucleotide polymorphism
LD: Linkage disequilibrium
GCTA: Genome-wide complex trait analysis
TDT: Transmission disequilibrium test
GO: Gene ontology
KEGG: Kyoto Encyclopaedia of Genes and Genomes
GEO: NCBI Gene Expression Omnibus database
MuTHER: Multiple Tissue Human Expression Resource project
HLCS: Holocarboxylase Synthetase, also known as Holocarboxylase Synthetase (Biotin-(Proprionyl-CoA-Carboxylase (ATP-Hydrolysing)) Ligase), Biotin Apo-Protein Ligase, EC 6.3.4.-, HCS
HLA-DRA: Major Histocompatibility Complex, Class II, DR Alpha, also known as MHC Class II Antigen DRA, HLA-DRA1, Histocompatibility Antigen HLA-DR Alpha
BICD2: BICD cargo adaptor 2, also known as SMALED2, bA526D8.1
VSIR: *V-set immunoregulatory receptor*, also known as V-Domain Ig Suppressor of T Cell Activation, Sisp-1, SISP1, Stress Induced Secreted Protein 1, Death Domain1alpha, DD1alpha, PP2135, B7-H5, VISTA, B7H5, GI24, c10orf54
