# Supplementary material for: A family-based genome-wide association study of chronic rhinosinusitis with nasal polyps implicates several genes in the disease pathogenesis
Source: PLoS One. 2017 Dec 18;12(12):e0185244. doi: 10.1371/journal.pone.0185244 (PMC5734761; doi:10.1371/journal.pone.0185244)
Supplement: S1 Table — (DOCX) [file pone.0185244.s001.docx]

**S1. List of target genes from the top 20 gene-sets in the INRICH analysis**

| **Source** | **Gene** |
| --- | --- |
| **GO + EMMAX** | ***COL16A1*** |
| **GO + EMMAX** | ***NDUFS5*** |
| **GO + EMMAX** | ***PPIH*** |
| **GO + EMMAX** | ***YBX1*** |
| **GO + EMMAX** | ***CLDN19*** |
| **GO + EMMAX** | ***LEPRE1*** |
| **GO + EMMAX** | ***ATG4C*** |
| **GO + EMMAX** | ***LEPR*** |
| **GO + EMMAX** | ***PDE4B*** |
| **GO + EMMAX** | ***GNG12*** |
| **GO + EMMAX** | ***SOAT1*** |
| **GO + EMMAX** | ***LAMB3*** |
| **GO + EMMAX** | ***CAPN2*** |
| **GO + EMMAX** | ***TP53BP2*** |
| **GO + EMMAX** | ***GNPAT*** |
| **GO + EMMAX** | ***CHRM3*** |
| **GO + EMMAX** | ***AKT3*** |
| **GO + EMMAX** | ***KIF3C*** |
| **GO + EMMAX** | ***SRSF7*** |
| **GO + EMMAX** | ***GEMIN6*** |
| **GO + EMMAX** | ***PRKCE*** |
| **GO + EMMAX** | ***CALM2*** |
| **GO + EMMAX** | ***ALS2*** |
| **GO + EMMAX** | ***CAB39*** |
| **GO + EMMAX** | ***RARB*** |
| **GO + EMMAX** | ***CACNA1D*** |
| **GO + EMMAX** | ***FHIT*** |
| **GO + EMMAX** | ***GSK3B*** |
| **GO + EMMAX** | ***CHST13*** |
| **GO + EMMAX** | ***DBR1*** |
| **GO + EMMAX** | ***B3GALNT1*** |
| **GO + EMMAX** | ***NMD3*** |
| **GO + EMMAX** | ***MECOM*** |
| **GO + EMMAX** | ***PRKCI*** |
| **GO + EMMAX** | ***KCNMB2*** |
| **GO + EMMAX** | ***USP13*** |
| **GO + EMMAX** | ***GBA3*** |
| **GO + EMMAX** | ***ANK2*** |
| **GO + EMMAX** | ***CAMK2D*** |
| **GO + EMMAX** | ***GPX3*** |
| **GO + EMMAX** | ***CPLX2*** |
| **GO + EMMAX** | ***E2F3*** |
| **GO + EMMAX** | ***ZFP57*** |
| **GO + EMMAX** | ***HLA-C*** |
| **GO + DFAM** | ***DDX39B*** |
| **GO + DFAM** | ***LTA*** |
| **GO + DFAM** | ***TNF*** |
| **GO + DFAM** | ***HLA-DRA*** |
| **GO + DFAM** | ***HLA-DRB5*** |
| **GO + DFAM** | ***HLA-DQA1*** |
| **GO + DFAM** | ***HLA-DQB1*** |
| **GO + DFAM** | ***PPARD*** |
| **GO + DFAM** | ***TTK*** |
| **GO + DFAM** | ***ATG5*** |
| **GO + DFAM** | ***STX7*** |
| **GO + DFAM** | ***UST*** |
| **GO + DFAM** | ***PDE10A*** |
| **GO + DFAM** | ***KIF25*** |
| **GO + DFAM** | ***AQP1*** |
| **GO + DFAM** | ***ADCY1*** |
| **GO + DFAM** | ***CDK6*** |
| **GO + DFAM** | ***PNPLA8*** |
| **GO + DFAM** | ***CHRM2*** |
| **GO + DFAM** | ***BRAF*** |
| **GO + DFAM** | ***PTPRN2*** |
| **GO + DFAM** | ***PPP2R2A*** |
| **GO + DFAM** | ***SFRP1*** |
| **GO + DFAM** | ***LYN*** |
| **GO + DFAM** | ***EYA1*** |
| **GO + DFAM** | ***ZFPM2*** |
| **GO + DFAM** | ***ST3GAL1*** |
| **GO + DFAM** | ***AGO2*** |
| **GO + DFAM** | ***CHMP5*** |
| **GO + DFAM** | ***BICD2*** |
| **GO + DFAM** | ***INIP*** |
| **GO + DFAM** | ***STXBP1*** |
| **GO + DFAM** | ***IL2RA*** |
| **GO + DFAM** | ***PRPF18*** |
| **GO + DFAM** | ***CUBN*** |
| **GO + DFAM** | ***PARD3*** |
| **GO + DFAM** | ***C10orf54*** |
| **GO + DFAM** | ***DDIT4*** |
| **GO + DFAM** | ***P4HA1*** |
| **GO + DFAM** | ***KIF20B*** |
| **GO + DFAM** | ***CPEB3*** |
| **GO + DFAM** | ***DOCK1*** |
| **KEGG + DFAM** | ***GTF2H1*** |
| **KEGG + DFAM** | ***BBOX1*** |
| **KEGG + DFAM** | ***WT1*** |
| **KEGG + DFAM** | ***SYT13*** |
| **KEGG + DFAM** | ***CAPN1*** |
| **KEGG + DFAM** | ***SIPA1*** |
| **KEGG + DFAM** | ***RELA*** |
| **KEGG + DFAM** | ***LRP5*** |
| **KEGG + DFAM** | ***DYNC2H1*** |
| **KEGG + DFAM** | ***PDGFD*** |
| **KEGG + DFAM** | ***DDX10*** |
| **KEGG + DFAM** | ***PRH1*** |
| **KEGG + DFAM** | ***PDE3A*** |
| **KEGG + DFAM** | ***GYS2*** |
| **KEGG + DFAM** | ***AQP2*** |
| **KEGG + DFAM** | ***SOAT2*** |
| **KEGG + DFAM** | ***RAP1B*** |
| **KEGG + DFAM** | ***NUP107*** |
| **KEGG + DFAM** | ***LYZ*** |
| **KEGG + DFAM** | ***FAM109A*** |
| **KEGG + EMMAX** | ***DDX51*** |
| **KEGG + EMMAX** | ***GTF2F2*** |
| **KEGG + EMMAX** | ***DCT*** |
| **KEGG + EMMAX** | ***LIG4*** |
| **KEGG + EMMAX** | ***GEMIN2*** |
| **KEGG + EMMAX** | ***PNN*** |
| **KEGG + EMMAX** | ***FERMT2*** |
| **KEGG + EMMAX** | ***SYT16*** |
| **KEGG + EMMAX** | ***JMJD7- PLA2G4B*** |
| **KEGG + EMMAX** | ***LIPC*** |
| **KEGG + EMMAX** | ***CCNB2*** |
| **KEGG + EMMAX** | ***MAP2K1*** |
| **KEGG + EMMAX** | ***AMFR*** |
| **KEGG + EMMAX** | ***NUDT21*** |
| **KEGG + EMMAX** | ***OGFOD1*** |
| **KEGG + EMMAX** | ***ULK2*** |
| **KEGG + EMMAX** | ***BRCA1*** |
| **KEGG + EMMAX** | ***XYLT2*** |
| **KEGG + EMMAX** | ***PRKCA*** |
| **KEGG + EMMAX** | ***EIF4A3*** |
| **KEGG + EMMAX** | ***RPTOR*** |
| **KEGG + EMMAX** | ***GNAL*** |
| **KEGG + EMMAX** | ***LSM7*** |
| **KEGG + EMMAX** | ***PDE4A*** |
| **KEGG + EMMAX** | ***PLCB1*** |
| **KEGG + EMMAX** | ***KIF16B*** |
| **KEGG + EMMAX** | ***NAPB*** |
| **KEGG + EMMAX** | ***MYL9*** |
| **KEGG + EMMAX** | ***TIAM1*** |
| **KEGG + EMMAX** | ***HLCS*** |
| **KEGG + EMMAX** | ***PLA2G3*** |
| **KEGG + EMMAX** | ***YWHAH*** |
| **KEGG + EMMAX** | ***SLC5A1*** |

Source: combination of pathways and methods that implicated this gene, GO=Gene Ontology, KEGG=Kyoto Encyclopedia of Genes and Genomes

Gene: HUGO gene ID
